# Sustainable Increase in Thermal Resistance of Window Construction: Experimental Verification and CFD Modelling of the Air Cavity Created by a Shutter

**DOI:** 10.3390/ma18122702

**Published:** 2025-06-09

**Authors:** Borys Basok, Volodymyr Novikov, Anatoliy Pavlenko, Hanna Koshlak, Svitlana Goncharuk, Oleksii Shmatok, Dmytro Davydenko

**Affiliations:** 1 Institute of Engineering Thermophysics, National Academy of Sciences of Ukraine, Marii Kapnist, 2a, 03057 Kyiv, Ukraine; borys.basok@gmail.com (B.B.); nvg52@i.ua (V.N.); goncharuk-s@ukr.net (S.G.); alexshok@ukr.net (O.S.);; 2 Department of Sanitary Engineering, Faculty of Environmental Engineering, Geomatics and Renewable Energy, Kielce University of Technology, Aleja Tysiąclecia Państwa Polskiego, 7, 25-314 Kielce, Poland; apavlenko@tu.kielce.pl

**Keywords:** double-glazed units, radiative heat transfer, thermal resistance, shutters, energy efficiency, sustainable building envelopes, CFD simulation, experimental analysis

## Abstract

This study investigates, both experimentally and theoretically, the impact of incorporating window shutters on the thermal resistance of double-glazed window units, employing computational fluid dynamics (CFD) modelling. The integration of shutters, whether installed internally or externally, introduces an additional air layer that significantly influences heat transfer between indoor and outdoor environments. This effect on the thermal performance of the transparent structure was analysed through experimental measurements under real operating conditions and numerical simulations involving fluid dynamics and energy equations for the air gaps, alongside heat conduction equations for the solid components. Fourth-kind boundary conditions, considering both radiative and conductive components of the total heat flux emanating from the building’s interior, were applied at the solid–gas interfaces. The simulation results, comparing heat transfer through double-glazed windows with and without shutters, demonstrate a substantial increase in thermal resistance, ranging from 2 to 2.5 times, upon shutter implementation. These findings underscore the effectiveness of employing shutters as a strategy to enhance the energy efficiency of windows and, consequently, the overall energy performance of buildings. This research contributes to the advancement of sustainable materials for engineering applications by providing insights into the optimisation of thermal performance in building envelopes.

## 1. Introduction

The accessibility of window glass for residential and other building construction increased significantly in the late 16th century, leading to its integration into contemporary window sashes [1,2]. Before this period, shutters served a multitude of functions, including protection against wind, rain, insects, birds, and intruders. In addition, shutters were employed for thermal insulation interior spaces during cold seasons and to mitigate the entry of solar radiation during warmer periods.

Glazed windows have since assumed comprehensive protective roles against environmental elements, including thermal insulation. However, fenestration systems remain critical areas of concern in building facades due to substantial specific heat losses in cold climates and excessive heat gains in warm climates. To address these issues, modern fenestration commonly incorporates energy-efficient single-, double-, and occasionally triple-glazed units. However, their thermal resistance remains significantly lower than that of insulated wall elements, which underscores the persistent challenge of enhancing the thermal performance. Research indicates that shutters can substantially reduce heat loss through window assemblies [3,4,5]. A standard double-glazed unit typically exhibits a thermal resistance of approximately *R_ter_* = 0.35 (m^2^K/W). The integration of shutters with glazed units can significantly reduce (by two to three times) thermal energy loss, particularly at night when solar gains are minimal.

A more precise and comprehensive approach to enhancing the thermal resistance necessitates combined computational and experimental investigations of the heat transfer processes through fenestration, analysing the impact of physical and design factors on heat exchange. These findings are crucial to the development and retrofitting of window systems that minimise heat loss and extend operational lifespan without compromising thermal properties. Enhancing window energy efficiency through measures such as shutter implementation can reduce thermal retrofitting costs and improve indoor environmental quality and occupant comfort.

The development of accurate methods for calculating heat transfer through transparent structures is a critical concern, as those currently employed in building thermal physics often rely on parameters that may not fully capture the influence of various factors. These factors include the number of glazing unit chambers, the spacing between glass surfaces, the thermophysical properties of the gaseous medium within these chambers, and the presence of coatings on glass surfaces designed to reduce radiative heat transfer. When shutters are incorporated, the geometric characteristics of the air cavity between the glazing unit and the shutters must also be considered.

In the research by [6,7], the focus is on evaluating the thermal resistance of the air cavity specifically between a glazing unit and an external shutter. This work utilizes empirical correlations for the convective heat transfer coefficient, incorporating the Rayleigh and Prandtl numbers. The radiative component of heat transfer is calculated using the Stefan–Boltzmann and Kirchhoff laws. However, the conductive component is not considered. The study demonstrates the dependence of the thermal resistance on the aspect ratio between the cavity height (*H*) and its width (*L*). Nevertheless, the conclusion regarding the temperature dependence of thermal resistance appears somewhat questionable.

The report [8] presents the results of a study conducted by the Scottish Energy Centre (SEC) at Edinburgh Napier University for the National Trust for Scotland to measure the U-value of two types of glazing insulation: shutters and films. The purpose of the study was to determine the benefits of these insulation measures when applied to an existing single-glazed window with a binding and box. The shutters were more effective in improving the thermal insulation properties of the existing single-glazed windows. Compared to a window without a shutter, the heat transfer resistance of the new design increases almost five times.

The problem of modernising existing window structures in order to increase their resistance to heat transfer is addressed in [9,10,11]. Similarly, Refs. [9,10] show that thermal shutters of the internal panel in the modernization of existing windows can significantly improve the thermal insulation characteristics of buildings, while maintaining the efficiency of existing glazing in providing natural light. The paper also argues that internal shutters achieve the best thermal performance when air infiltration into the space between the window and the shutter is minimised. This minimises the risk of liquid condensation behind thermal shutters.

The enhancement of thermal resistance in window assemblies through the incorporation of additional elements has been extensively investigated [12,13]. These studies share a common methodological approach employing software tools such as Berkeley Lab WINDOW v. 7 to analyse heat transfer processes between the external environment and the building’s interior through window systems equipped with these additional components. In [14], a single-glazed unit with internally installed blinds is examined. The findings reveal that the spacing between the blinds and the glazing significantly impacts the overall heat transfer coefficient (U-value). Considering economic feasibility, the author recommends using double-glazed windows with a 30 mm distance from the blinds. This configuration achieves a U-value of 2.1 W/m^2^K, corresponding to a thermal resistance (*R_term_*) of 0.47 m^2^K/W.

In [15], the applicability of ASHRAE 90.1 software to simulate heat transfer within complex fenestration systems is examined. This study focusses on the modelling of glazing systems integrated with various shading devices, including shades, blinds, curtains, roller shutters, and insect screens, employing internally mounted blinds as a specific case. The research elucidates inherent limitations within the software, notably a reduction in accuracy when the distance between glazing and shading elements exceeds 10 mm.

Thermal shutters offer a viable solution to significantly reducing heat loss through windows and enhancing the overall energy performance of buildings [16,17]. These studies employ computational modelling to investigate the impact of advanced insulation materials, specifically vacuum insulation panels, on improving the thermal characteristics of window assemblies equipped with shutters fabricated from these panels. The results demonstrate a substantial reduction in heat loss through windows when using vacuum insulation panels as internal thermal shutters. In particular, the thermal insulation performance of windows with vacuum insulation panel shutters is shown to be 2.5 times superior to that of the same window without shutters. This improvement is attributed to the relatively large thickness of the vacuum panel (50 mm) and its exceptionally low thermal conductivity (0.005–0.008 W/(m·K)).

A comprehensive review of strategies to improve fenestration energy efficiency is presented in [18,19,20,21,22]. These studies systematically evaluate the efficacy of various interventions, including curtains, drapes, blinds, screens, and shutters, based on a variety of performance metrics. These metrics encompass heat transfer reduction, solar heat gain modulation, daylighting provision, thermal comfort maintenance, condensation risk mitigation, air leakage control, economic feasibility, operational convenience, privacy provision, and aesthetic integration. Utilising data derived from the scholarly literature, manufacturer specifications, and computational tools such as WINDOW and THERM version 7.8.77, the review concludes that internal applications of energy efficiency enhancements are predominantly effective in conserving interior thermal energy, while external applications primarily serve to regulate solar radiative influx through building facade apertures.

It is pertinent to note that most prior investigations have predominantly focused on the individual components of window assemblies integrated into building facades. However, the progressive advancement of computational technology has enabled a comprehensive analysis of multifaceted factors and design parameters that influence heat transfer through fenestration systems through computational fluid dynamics (CFD) modelling. This methodology facilitates the numerical solution of equation systems that govern fluid dynamics within the air cavities of window assemblies, heat exchange between the components of the glazing unit, and thermal transfer from the window to its ambient environment. Contrary to the assertion in [5], which posits a static air condition within the climatic chamber during single-glazed unit heat transfer experiments, fluid motion in proximity to component parts of the window assembly is invariably present. This motion, driven by natural convection, arises from temperature gradients between interior and exterior environments. This observation is corroborated by findings presented in [23,24,25].

Considering the current state of computational technology, the aforementioned factors can be investigated concurrently through CFD modelling, which numerically solves the equation systems governing fluid dynamics within window assembly air cavities, heat exchange between glazing unit components, and heat transfer from the window to the environment. Fluid motion is primarily driven by natural convection, arising from temperature differences between the interior and exterior environments.

*Shutter designs and materials for their manufacture.* Considering the construction of stationary shutters, the following should be distinguished [26]:

- Louvred shutters, which are the most common types of shutters; fixed louvres or slats are angled to filter light, provide ventilation and add aesthetic appeal.
- Raised panel shutters are stationary shutters that have a classic flat middle part with bevelled edges, which gives the facade rigidity.
- Shaker-style shutters include a flat component with vertical shallow seams spaced equally apart; this fixed construction can also be known as shaker, country, cottage, frame board, and batten or craftsman shutters.
- Flat panel shutters are more modern in appearance and are often installed on modern facades to improve the overall aesthetic appearance of the building.
- Combined shutters combine the design features and advantages of a louvred shutter and a lift panel.
- The board and batten shutters have a simple design and appearance that gives a modern urban building the look of a rural farmhouse.
- Bahama shutters are characterised by a design in which each panel is hinged at the top of the window, allowing it to project outward from the bottom; these shutters frequently incorporate multiple horizontal rows of louvers. This architectural feature is predominantly observed in southern regions.

The aforementioned list of shutters primarily encompasses stationary designs, typically installed externally. These stationary shutters are fabricated predominantly from materials such as wood, polyvinyl chloride (PVC), medium density fibreboard (MDF) composites, vinyl, and other similar substances. In contemporary applications, the term “shutters” also extends to include blinds and roller shutters, or roller blinds. Blinds are primarily utilised for solar radiation control, whereas roller shutters fulfil the comprehensive functions of traditional shutters. The slats or aluminium profiles, which constitute the primary components of roller shutters, are constructed from thin-walled aluminium, often filled with polyurethane foam. Modern external roller shutters represent a more contemporary and aesthetically pleasing alternative to stationary grilles, wooden shutters, and metal shutters. Equipped with user-friendly operating mechanisms, roller shutters can be easily raised, providing occupants with unobstructed views of the external environment at any time.

The primary objective of this study is to comprehensively evaluate and quantify the improvement of thermal resistance in double-glazed windows through the integration of shutters. This research aims to achieve this by employing a combined methodology of on-site experimental measurements and computational fluid dynamics (CFD) simulations. Specifically, the study seeks to

- determine the impact of both internal and external shutter placement on the overall heat transfer characteristics of double-glazed window assemblies;
- Analyse the fluid dynamics and energy equations within the inter-pane air cavities and the heat conduction within solid components of the window structure under realistic operating conditions;
- Quantify the increase in thermal resistance achieved by incorporating shutters, and assess the effectiveness of shutters as a strategy for improving the energy efficiency of windows and building envelopes;
- Investigate the influence of various design and physical factors, including the geometric characteristics of the air cavity between the glazing unit and shutters, on the heat transfer processes;
- Validate the numerical simulations against experimental data to ensure the accuracy and reliability of the findings;
- Provide practical recommendations for the selection and implementation of shutters to optimise thermal performance in both new and retrofitted window systems.

The purpose of the article is to increase the thermal resistance of an existing translucent structure by using shutters; in our case, this is an additional sequential installation in the window opening of the wall of stationary vertical screens made of transparent solid polycarbonate. Screens are hermetically installed in relation to the wall. The distance between the screens is not less than 3–4 cm, as our previous studies have shown. That is, the idea was to divide the window air niche in the wall into several isolated vertical air blocks, which are similar to the air glass chambers of the double-glazed window itself.

## 2. Materials and Methods

For theoretical modelling, basic equations of mathematical physics for the laws of conservation of mass, momentum, and energy were used. External solar insolation, the possible condensation of water vapour, and the dependence of the thermophysical properties of window materials on temperature were not taken into account, and an ideal gas was used as air. Therefore, the model was incomplete.

However, the model was adequate, because of the following:

1. For comparison with our own experiment, evening and nighttime data were used, when insolation (direct and diffuse) was absent.
2. There were no personnel or other disturbances in the room.
3. When verifying the model, the calculations for a single two-chamber window with a low-emissivity coating coincided with the data from our own independent experiment and with the Ukrainian regulatory standard for the thermal resistance of this window within ±0.01 m^2^K/W (1.5%).

In general, we note that the solution of the system of partial differential equations for heat transfer was performed using a finite volume numerical method of second-order accuracy of approximation in spatial coordinates. This is a general parameter of the accuracy of obtaining numerical results.

To examine the intricate heat transfer phenomena occurring within double-glazed windows and integrated blind systems, a comprehensive computational fluid dynamics (CFD) modelling strategy was developed. The overarching objective of this research is to provide a detailed physical understanding of the mechanisms governing heat transfer resistance in these translucent enclosures. Specifically, the methodology involved the formulation of the CFD modelling problem, numerical simulation of airflow and heat transfer, and physical substantiation of key factors.

The STAR-CCM+ 2302 computer package was used for the calculations.

**Figure 1:**
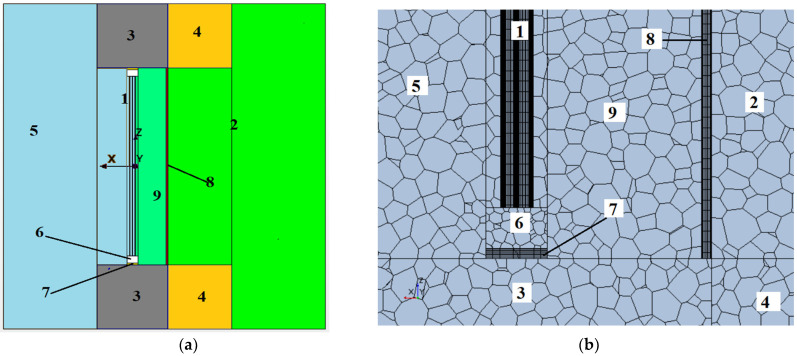
Representation of the computational model. (**a**) Three-dimensional geometric domain illustrating the window system, featuring the following: 1—double chamber glass unit 4M1-10-4M1-10-4M1 (1.06 × 0.642 m), 2—external air (*T_out_* = −10 °C), 3—brick wall (0.38 m), 4—basalt thermal insulation (0.34 m), 5—internal air (*T_in_* = 20 °C), 6—three chamber frame (0.04 × 0.06 m), 7—polyurethane foam (0.01 m), 8—polyurethane shutter (0.01 m), and 9—air layer between glass and shutter. (**b**) Cross-sectional view of the computational grid, containing more than 1,025,000 cells.

A three-dimensional computational domain, as illustrated in Figure 1, was established for numerical analyses. The geometric configuration was defined to accurately represent the window structure, including the double-glazed unit and the integrated blinds. To provide a clear visualisation of the model’s internal structure, a cross-sectional plane was defined. This plane, perpendicular to the surfaces of the glass, walls, and blinds, traverses the midplane of the insulating glass unit, where the y-coordinate is set to zero.

The geometric model represents a section of an energy-efficient building facade, which incorporates a window aperture, as developed at the IET of the National Academy of Sciences of Ukraine. The origin of the coordinate system is positioned at the centroid of the exterior glass surface (Figure 1). The three-chamber insulating glass unit frame profile is modelled as a rectangular solid with homogenised material properties, representing PVC, air, and a steel reinforcement bar. The window system features a roller shutter with thin-walled polyurethane-filled lamellae. Three distinct CFD model configurations were developed: (1) an external shutter positioned at the facade wall level, (2) a baseline configuration without shutters, and (3) an internal shutter situated at the building wall level.

### 2.1. Physical Formulation of the Problem

The physical formulation of the problem concerns the analysis of steady-state thermal transport within a composite system, comprising an indoor air domain maintained at 20 °C, a double-glazed window unit, a three-chamber frame structure, a section of the building facade, and an external ambient air domain at −10 °C. The heat transfer mechanism, driven by the imposed temperature difference, is characterised by the coupled phenomena of convection, conduction, and radiation. Natural convective flows within the air domains result in the formation of hydrodynamic and thermal boundary layers at solid–fluid interfaces. The radiative component of the heat flux accounts for long-wavelength thermal radiation. The momentum and energy transport processes within the system are described by a three-dimensional system of partial differential equations, including the continuity equation, the momentum transport equations (Navier–Stokes), the energy transport equation for the fluid phase, and the Fourier heat conduction equation for the solid phases. The ideal gas equation of state is employed to model the thermodynamic properties of air.

On the solid–air and solid–solid interface surfaces, interface conditions are set that consider conductive heat exchange between solid surfaces and air, as well as radiative heat exchange between solid surfaces. The above system of equations with limit conditions is solved by a numerical method. The method of the numerical solution of this problem is discussed in detail in [27,28]. According to the results of the numerical solution of the system of equations, the velocity, pressure, and temperature fields in the glass, in the air of the window niche, and in the gas layers of the insulating glass unit are determined.

### 2.2. Methodology for Experimental Investigations Under Real-World Meteorological Conditions

**Figure 2:**
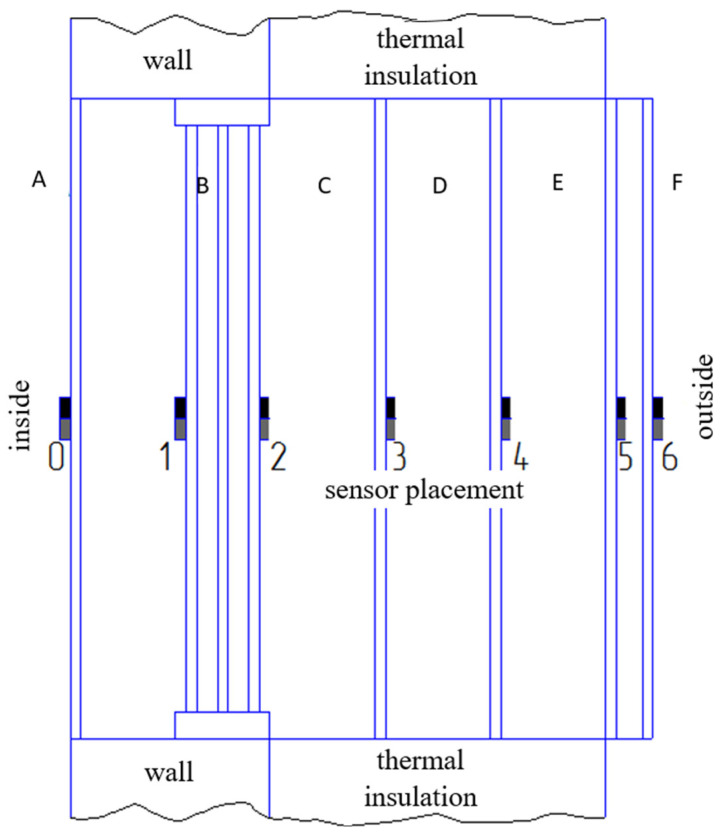
Schematic diagram of the window block: window opening with dual niches (internal and external) + window + shutter system with sensor locations. Designations: B—double-chamber window; A, C, D, and F—stationary shutters; and E—double-leaf opening shutter.

Experimental investigations of shutter influence on the thermal resistance of a 4M1i-10-4M1-10-4M1 window assembly with low-emissivity coating were conducted using a multilayered block of framed panels. These panels consisted of 2.0 mm thick transparent solid polycarbonate and 4.0 mm thick transparent cellular polycarbonate. The panels were installed at various depths within the window wall aperture, on both the interior and exterior sides. Figure 2 illustrates a schematic cross-sectional vertical view (perpendicular to the facade wall) of the complete assembly, indicating the positions of temperature and heat flux sensors.

The experimental procedure involved sequential measurements of various shutter block configurations, each subsequent measurement incorporating an additional polycarbonate panel. The configurations were as follows: variant 1—window B (all designations are consistent with Figure 2); variant 2—window B with shutter C; variant 3—window B with shutters C and D; variant 4—window B with shutters C, D, and double-leaf opening shutter E; variant 5—window B with shutters C–F; and variant 6—internal shutter A with window B and shutters C, D, E, and F. Shutters designated A, C–F were installed in a fixed configuration within the opening. Shutters A and F were constructed from 4.0 mm thick cellular polycarbonate, while shutter E comprised two outward-opening leaves.

The polycarbonate panels for shutters C–E were installed with protective transport films intact. The experimental apparatus utilised a westward-oriented window on the facade of an IET experimental passive zero-energy building.

## 3. Results

### 3.1. Analysis of the Results of Numerical Studies

**Figure 3:**
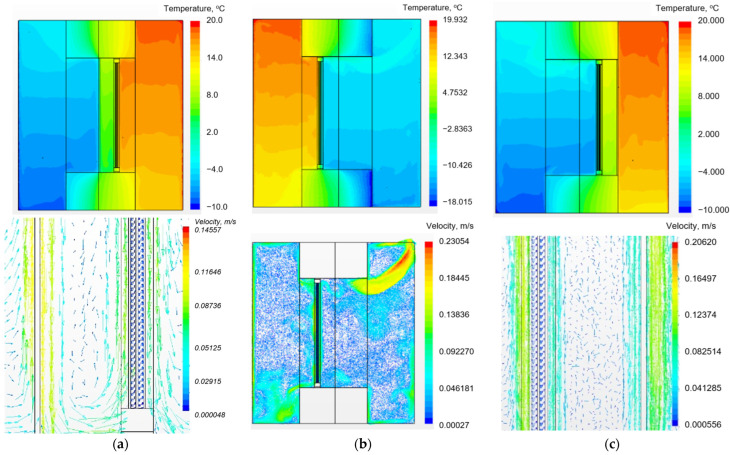
Numerical results depicting the temperature and velocity distributions for the analysed scenarios: (**a**)—exterior shutters integrated into the facade wall plane; (**b**)—baseline window configuration without shutters; and (**c**)—interior shutters located at the room wall interface.

A comparative study was carried out to determine the effects of shutters on the heat transfer performance of the fenestration system illustrated in Figure 1, which accounts for the influence of the components of the adjacent facade. Analyses were conducted for configurations with and without shutters, with shutter placement varied between the interior and exterior sides. The resulting velocity and temperature distributions, derived from 3D CFD simulations across vertical planes within the computational domain, are presented in Figure 3 for the three specified scenarios. Furthermore, beyond standard numerical simulations (V1 and V3), additional computations were executed for a double-chamber insulating glass unit with the configuration 4M1i-10-4M1-10-4M1 (interior to exterior). This glazing configuration incorporates an i-coating on the inner glass surface, which significantly reduces surface emissions to ε = 0.2, compared to ε = 0.93 for conventional glass. This reduced emissivity effectively mitigates the radiative component of the heat flux emanating from the interior, consequently improving the overall thermal resistance of the window structure.

The airflow visualisation presented in Figure 3 reveals that the vertical air velocity profiles within the inter-pane cavities of the double-glazed unit are analogous to those thoroughly documented in [29]. A distinct recirculating flow regime is established within the airspace bounded by the shutters and the double-glazed unit, which features the development of boundary layers along the respective surfaces. The central portion of this airspace is characterised by a region of stagnant air.

**Figure 4:**
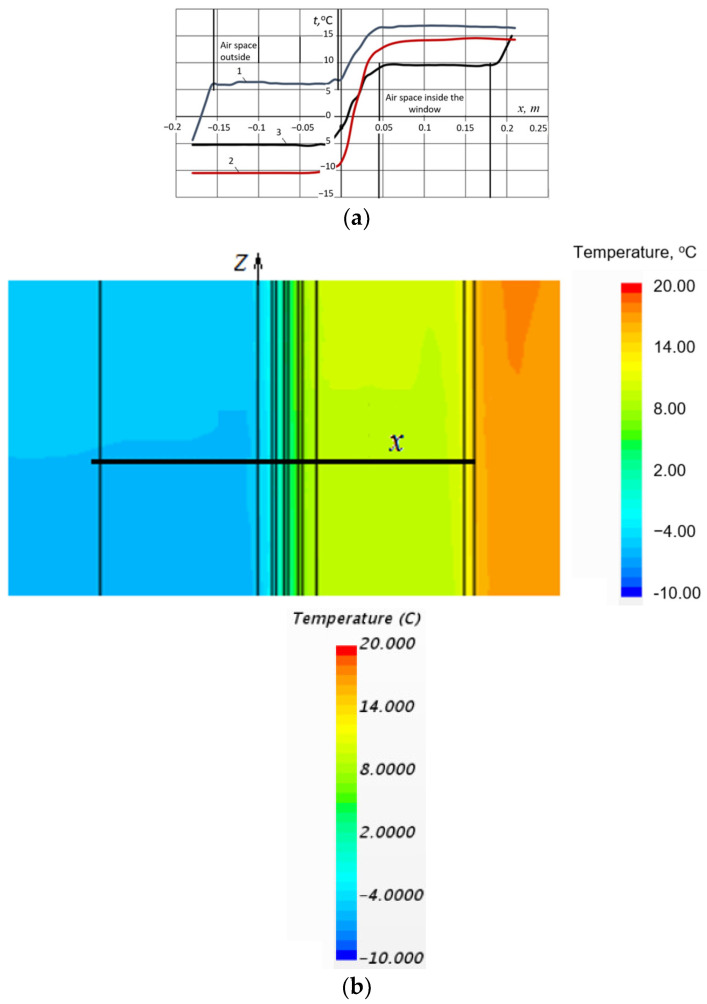
Window assembly temperature profiles. (**a**) Temperature distributions along the thickness of the window assembly: 1—configuration with external shutters; 2—configuration without shutters; and 3—configuration with internal shutters. (**b**) Spatial location of the line used for temperature profile extraction.

Figure 4a elucidates the temperature distributions across the thickness of the computational domain for the three distinct configurations under investigation. To provide a comprehensive understanding of the thermal profiles, temperature data were extracted along a linear trajectory that traverses the geometric centre of the computational model, extending from the exterior facade to the interior wall boundary, as depicted in Figure 4b.

The analysis of air circulation velocities within the inter-glass spaces of the insulating glass unit revealed exceptionally low magnitudes (0.02 m/s). This observation strongly suggests that convective heat transfer plays a negligible role in the overall thermal transport mechanism within these regions. Consequently, the temperature profile across the gas layers of the double-glazed unit manifests a near-linear characteristic (Figure 4a, curve 2), a hallmark signature of a conduction-dominated heat transfer regime. This linearity underscores the dominance of conductive heat transfer over convective contributions within the sealed glass layers.

Furthermore, within the central airspace formed between the shutters and the double-glazed unit, a distinct region of stagnant air was observed characterised by a nearly uniform temperature distribution (Figure 4a, curves 1 and 3). The observed temperature uniformity suggests the absence of substantial air movement, consequently implying negligible convective heat transfer within this enclosed airspace. This stagnant air layer effectively functions as an additional insulating medium, thereby enhancing the overall thermal resistance of the system.

The observed temperature profiles, therefore, provide compelling evidence of the dominant role of conduction in heat transfer through the multilayered glazing system, with minimal contributions from convection, especially within the sealed inter-glass spaces. The stagnant air region between the shutters and the double-glazed unit further enhances the thermal insulation properties of the system, showcasing the importance of air layer configurations in thermal management.

**Figure 5:**
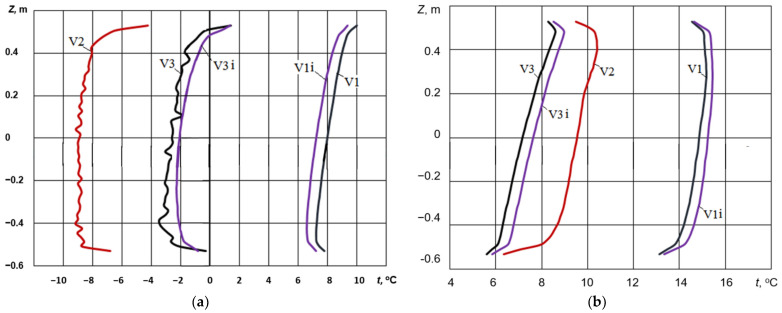
Temperature distribution along the vertical centreline of the glass surfaces: (**a**) outer surface and (**b**) inner surface. V1, V2, and V3 correspond to the simulation scenarios presented in Figure 2; the designation i is the variant with i-coating on the inner surface of the glass.

The resulting flow and thermal fields are comparable to those observed in a configuration employing two serially arranged double-glazed units [30], which implies that radiative heat transfer is the primary mechanism governing the thermal exchange between the double-glazed unit and the shutters. Figure 5 presents the temperature distribution along vertical lines intersecting the geometric centres of the glass surfaces interfacing with the ambient environment (a) and the air in the room (b).

The data presented in Figure 5 demonstrate that the implementation of shutters has a profound effect on the temperature distribution profiles across the glass surfaces of the double-glazed unit. In particular, shutters effectively attenuate the temperature gradient across the unit. The average temperature differentials between the external and internal glass surfaces were quantified as follows: Δ*t* = 5.8 °C for configuration V1, Δ*t* = 16.5 °C for configuration V2, and Δ*t* = 7.9 °C for configuration V3. The deployment of external shutters provides a thermal barrier against the exterior environment, resulting in an elevation of temperature on both the external and internal glass surfaces, with both temperatures remaining above zero.

**Figure 6:**
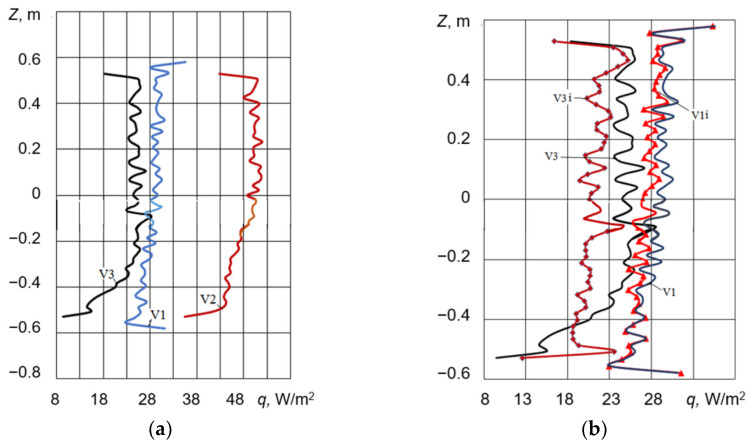
Heat flux density at the central region of external glass surfaces: (**a**) for various window configurations and (**b**) comparison of heat flux for window assemblies with and without i-coating.

**Figure 7:**
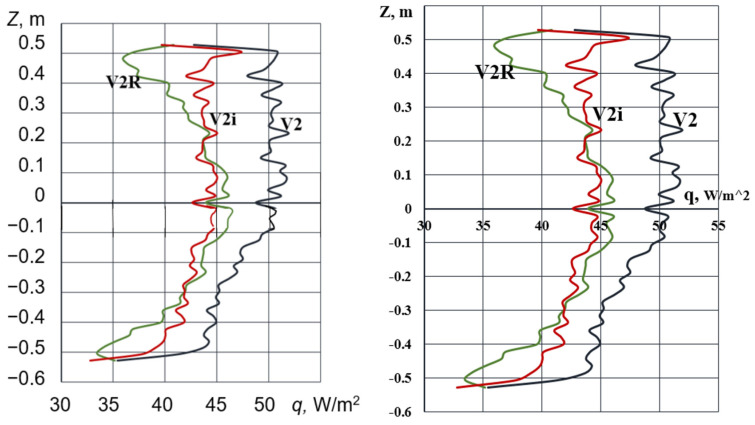
Heat flux density at the central region of the external surface of the unshuttered window assembly: V2—heat flux density of a standard double-glazed unit; V2i—double-glazed unit with an i-coating on one glass pane; and V2R—radiative heat flux density of the standard double-glazed unit.

The magnitude of heat flux represents a crucial thermophysical parameter for any fenestration system, especially considering the inherent vulnerability of windows as primary conduits for heat loss in building envelopes. Figure 6 and Figure 7 present the heat flux density distributions at the central area of the glass surfaces exposed to the external environment. As shown in Figure 6a, the configuration featuring interior shutters (V3) exhibits the lowest heat flux towards the external environment, thereby indicating the most energy-efficient design among the studied cases.

Figure 6b provides a comparative analysis of the heat flux density values for configurations V1 and V3, contrasting the results obtained with and without the implementation of an i-coating on the glass surface of the double-glazed unit that is proximate to the interior environment.

It should be noted that the application of the i-coating demonstrates a marginal influence on the overall heat loss through the shuttered window assembly. Specifically, for configuration V1, the heat flux density with the i-coating is reduced by approximately 3% on average. For configuration V3, the corresponding reduction is observed to be 14%.

**Figure 8:**
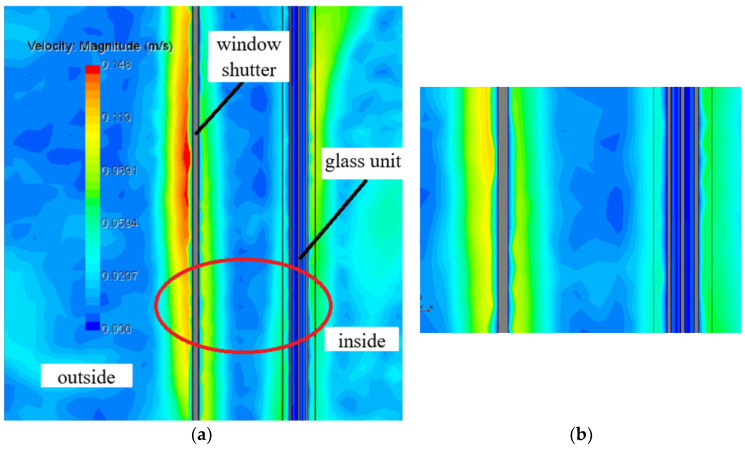
Air velocity field in the window assembly with external shutters: (**a**) unstable boundary layer on air-contacting surfaces and (**b**) enlarged detail from (**a**).

For the unshuttered window assembly depicted in Figure 7, the implementation of an i-coating results in an 11% reduction in heat loss (curve V2i, Figure 7). To provide context, the radiative component of the heat flux density to the ambient environment is presented, revealing that it constitutes over 60% of the total heat loss. The observed oscillations in the heat flux density along the vertical line on the external window surface are attributed to the inherent instability of the natural convection boundary layer forming on these surfaces (Figure 8).

An essential metric for assessing the thermal insulation properties of building facade elements, specifically window assemblies, is thermal resistance. This parameter is quantified through the application of the subsequent computational formula:

*R_ter_* = (*T_in_* − *T_out_*)/*q_out_*

where *T_in_* represents the temperature of the glass surface interfacing with the indoor environment, *T_out_* denotes the temperature of the glass surface exposed to the outdoor environment, and *q_out_* signifies the heat flux density transmitted through the double-glazed unit to the surrounding environment.

Based on the numerical simulations, thermal resistance was determined for the three considered configurations, including the window assembly with shutters. In the latter case, the exterior shutter surface temperature was used in place of the exterior glass surface temperature in the thermal resistance formula. It is noted that the heat transfer resistance calculations were performed using area-averaged values. Table 1 presents the thermal resistance values for all of the investigated configurations.

### 3.2. Results of Experimental Analysis Under Real Meteorological Conditions

**Figure 9:**
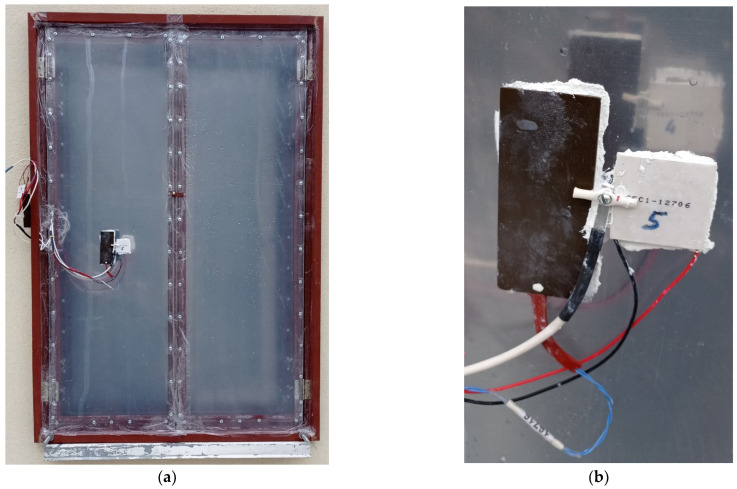
Photographic view of the window assembly: (**a**) frontal view of the hinged shutter E and (**b**) close-up of heat flux sensors (dark—thermoelectric, IET NAS of Ukraine, Ukraine; white—semiconductor China) and a temperature sensor CRT (copper resistance thermometer) affixed with thermal paste.

The following analysis details the findings of experimental studies conducted in authentic meteorological environments, with a particular emphasis on the thermal resistance of various window and shutter configurations. Figure 9 provides a visual representation of the experimental setup, featuring a frontal view of the hinged shutter E (Figure 9a) and a detailed close-up of the heat flux and temperature sensors affixed to the window assembly (Figure 9b). In the background of (b), the sensors of shutter D and the silhouette of the shutter C sensors are visible.

**Figure 10:**
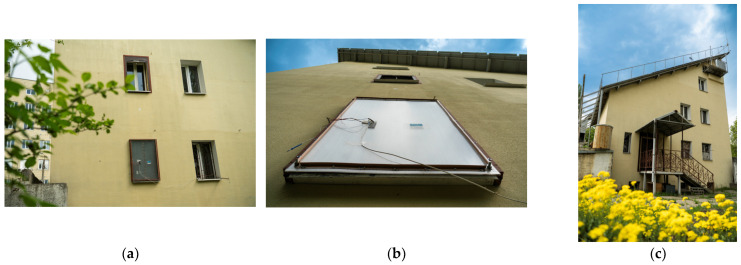
Exterior views of the passive house with installed shutter: (**a**) fragments of the eastern facade; (**b**) bottom view of the installed shutter; and (**c**) western façade of the passive house at the Institute of Engineering Thermophysics, National Academy of Sciences of Ukraine, Kyiv.

The location of the window structure with shutters was on the second floor of the eastern facade of the experimental passive house of the institute in Kyiv (hereafter referred to as the passive house), Figure 10a.

**Figure 11:**
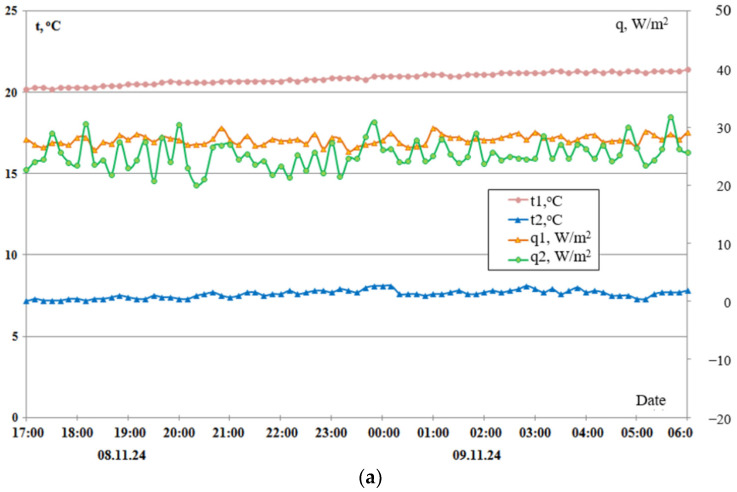

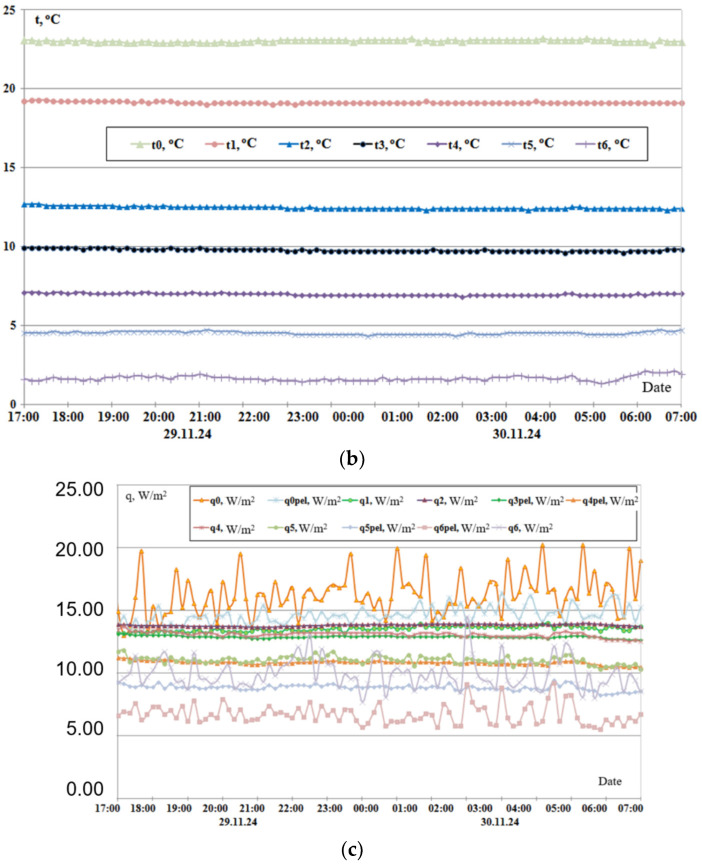
Experimental data of nighttime temperatures and heat fluxes at key locations within the window block with shutters, for different installation configurations: (**a**) variant 1, (**b**,**c**) variant 6.

A series of graphs depicting experimental data for six distinct window block configurations, incrementally implemented from variants 1 to 6, are presented in Figure 11. Thermal resistance calculations were performed using quasi-steady-state measurements obtained exclusively during nighttime hours, ensuring the absence of solar insolation and personnel influence. The comprehensive methodology employed for measurement execution and data processing is thoroughly described in [31].

A summary of the thermal resistance data, extracted from the experimental graphs depicted in Figure 11, is provided in Table 2.

### 3.3. Thermophysics of Heat Loss to the Environment

For a better understanding of the thermophysical processes occurring in such a complex system of barriers and the spread of heat from the building premises to the environment, let us consider in more detail the main heat flows, namely from the premises to the internal shutter (designation “0”, flow *q*_0_), then the flow *q*_1_ to the window, then the flow *q*_2_ from the window to the external shutter system, and then the flow *q*_6_ to the environment. Initially, we will outline the methodology employed for conducting measurements and processing the resultant data.

The temperature was measured by platinum (sputtering) and copper resistance thermometers with random errors of 0.05 °C and 0.1 °C, respectively. The specific heat flow was measured by multi-junction battery thermocouple sensors of our own production (up to five conductor junctions per square millimeter) and semiconductor sensors of the Peltier type (China), configured to use the Seebeck effect and calibrated on a special metrological stand. Consequently, the random measurement errors were 0.5 W/m^2^ and 1 W/m^2^. The sensitivity of each of the sensors is quite high—0.2 W/m^2^. The thermocouple sensor of the specific heat flux (dark brown, on the left in Figure 9b) has a size of 0.04 × 0.08 × 0.002 m, the semiconductor Peltier sensor (white, on the right in Figure 9b) has a size of 0.04 × 0.04 × 0.004 m, and both were placed on a layer of thermally conductive paste (white colour).

**Figure 12:**
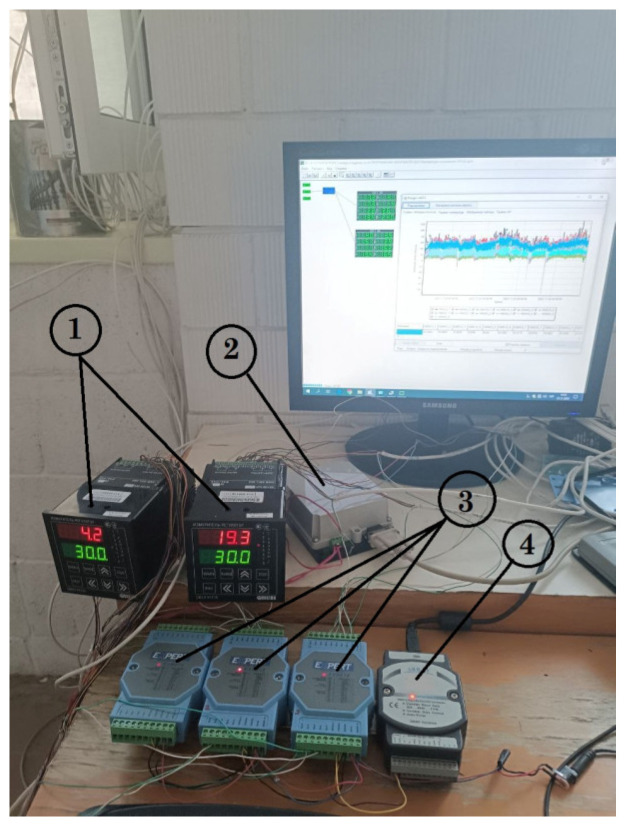
Experimental data acquisition and processing unit components: 1—temperature measurement and control device; 2—measuring signal converter; 3—UKT-38 temperature measurement and control device; and 4—“Expert” analogue-to-digital converter.

All experimental data were automatically converted into digital format, recorded, and archived in computer memory without researcher intervention. Then, statistical data processing was performed. A photograph of the main components of the automated measuring system is shown in Figure 12.

We note in passing that experimental studies were conducted for a two-chamber window with one low-emission coating, which is installed in a real climate in a full-scale (4.5 floors, total area of 300 square meters) experimental passive house of the Institute of Engineering Thermophysics of the National Academy of Sciences of Ukraine, Figure 10. In addition, in the administrative building of the institute there is a special testing ground with 20 energy-efficient windows of various designs and made of different materials. For a period of 15 years, the thermophysical aspects of heat loss and heat gain, encompassing the degradation (aging) of thermal parameters in windows and other building structures, have been meticulously investigated at that location. Some experience in conducting precision experiments has been gained, and the corresponding results have been obtained.

**Figure 13:**
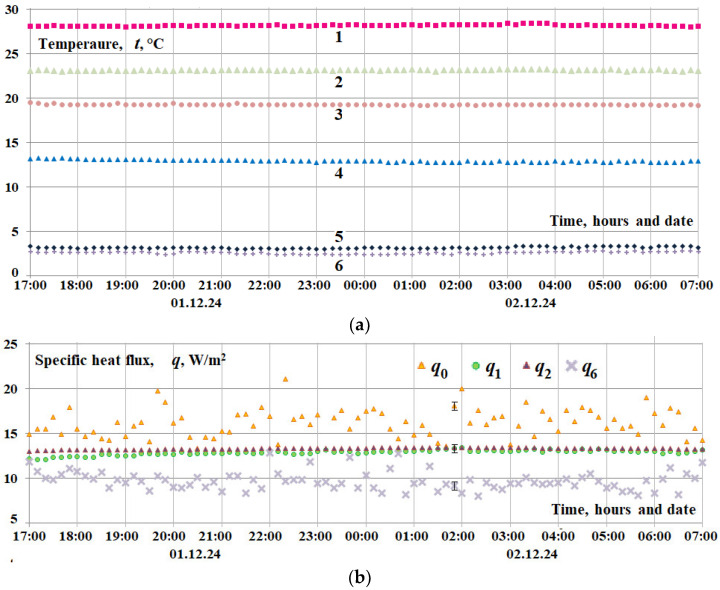
Indicators of thermal interaction of the window structure with the room, facade wall, and the environment during the dark period of a winter day. (**a**) Temperature designation. (**b**) Specific heat flux: 1—air in the room; 2—on the inner surface of the first shutter from the room side; 3—on the inner surface of the window glass from the room side; 4—on the outer surface of the window glass from the environment side; 5—on the outer surface of the last shutter from the environment side; and 6—environment.

Consequently, experimental studies of thermal characteristics were carried out using heat flux converters (HFCs) and temperature sensors connected to secondary devices: eight-channel analogue-to-digital converters, “Expert” (position 3 in Figure 12), for the HFCs and temperature measurement and control devices, UKT-38 (position 1 in Figure 12). Subsequently, a signal converter (USB to RS-232/422/485) facilitated the connection between the secondary devices and a personal computer, with automatic management of data transmission direction (UKT-38: position 2, Figure 12; “Expert”: position 4, Figure 12). Consequently, specialised software automatically logged, digitised, and stored the experimental temperature and heat flux data for real-time monitoring and further analysis. Figure 13 shows experimental data on temperature measurements and specific heat losses in characteristic places of the window structure during the dark period of the transition from 1 to 2 December 2025, i.e., in the evening, at night, and very early in the morning.

It should be noted that the random errors of temperature measurements are within the size of different dot labels for all points in Figure 13, and for heat flux, within two dot label sizes. The vertical range of random error for heat flux can be seen in the time domain 02.00. This night transition turned out to be very successful because the night ambient temperature was constant, which does not often happen on winter nights due to the weather in Kyiv. The dynamics of all temperatures were constant, that is, all temperatures (designations 1–6. Figure 13) were constant within the measurement errors. The average temperature values during the time interval 17.00–0.00–07.00 in °C were as follows: temperature inside the room *t*_1_ = 28.2 ± 0.1; on the inner surface of the first shutter *t*_2_ = 23.2 ± 0.1; on the inner surface of the double-glazed window *t*_3_ = 19.3 ± 0.1; on the outer surface of the double-glazed window *t*_4_ = 12.9 ± 0.1; on the outer surface of the last shutter *t*_5_ = 3.2 ± 0.1; and in the environment *t*_6_ = 2.6 ± 0.1.

The average specific heat fluxes were as follows: flow from the room to the first inner shutter, *q*_0avg_ = 16.6 ± 1.3 W/m^2^—this flux increased slightly with time; flow to the inner surface of the window, *q*_1avg_ = 12.9 ± 0.2 W/m^2^; flow from the outer surface of the window, *q*_2avg_ = 13.3 ± 0.1 W/m^2^; flow from the outer surface of the last shutter to the environment, *q*_6avg_ = 9.7 ± 0.8 W/m2—this flux decreased slightly with time. With a temperature difference between the room and the environment of 25.6 °C, the specific heat fluxes of heat loss were not large in magnitude and were in the range of 18 ÷ 8 W/m^2^. The difference in heat fluxes on average ranged from 5 to 10 W/m^2^. The above indicators indicate the high energy efficiency of the proposed window design.

**Figure 14:**
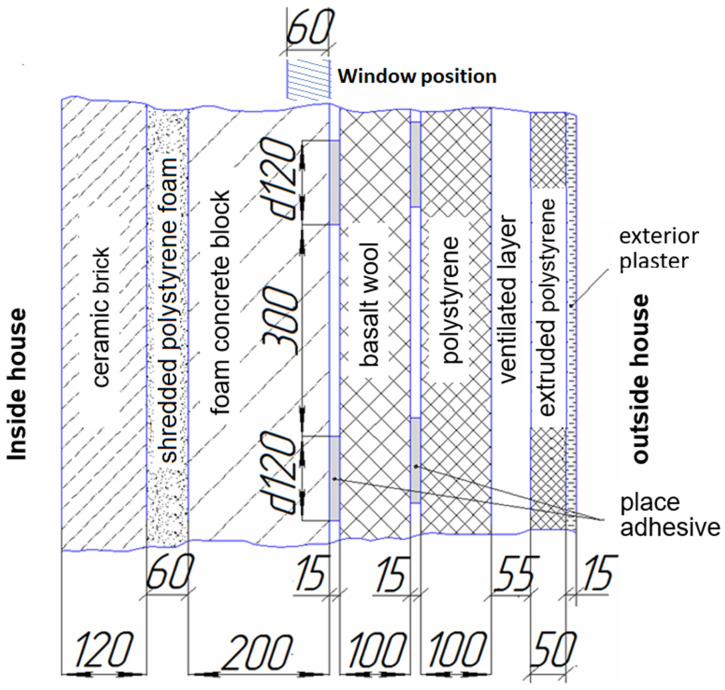
Multi-layered passive house exterior wall (dimensions in mm). Total thickness: 74–75 cm.

The heat flows at the entrance and exit of the double-chamber window are the same within the error limits. This means that there are no lateral heat drains into the wall structure of the window opening. The reason for this is two important circumstances. First, the window is not simply mounted in a niche, but is inserted into an intermediate special thermal insulation insert in the form of a plastic empty (or filled with polyurethane foam) rectangular pipe with a width equal to the thickness of the window. Only then is this joint mounted in the window opening. Another reason is the transverse structure of the wall (Figure 14), especially the external thermal insulation layer with a thickness of 34–35 cm. The double-chamber, double-glazed window unit was installed such that its external surface (in direct contact with the environment) was flush with the outermost plane of the base facade wall, prior to the application of external insulation. Consequently, the entire external window reveal was insulated along its perimeter with a multi-layer thermal insulation material measuring approximately 34 to 35 cm in thickness.

It is noteworthy that the heat flux variations on the inner surface exhibit a greater amplitude than those observed on the outer surface, a trend that typically contradicts conventional expectations. These fluctuations are due to the convective component of the heat flux; traditionally it is smaller in the room, where there are fewer air disturbances, and larger on the side of the environment, where wind flows of a certain height distribution of speed operate. On the night of 1 December to 2 December, there was a wind from the east in Kyiv with a fairly low speed, at a height of 10 m; it only sometimes reached 1 m/s. This did not contribute to the impact of speed pulsations on the facades of buildings, especially in the conditions of the dense construction of the institute’s territory. On the contrary, the heating system on the premises operated on the basis of an electric heater, which automatically maintained the set temperature, episodically turning on or off the power supply, which disturbed the aerodynamic situation.

## 4. Discussion

Contemporary Ukrainian building regulations [32] stipulate a thermal resistance requirement of 0.90 m^2^K/W for window assemblies. The data presented in Table 2 substantiates that the deployment of four unadorned shutters, equidistantly spaced at approximately 9 cm within the window opening niche, effectively doubles the thermal resistance. This enhancement is achieved through a simplistic and financially prudent methodology. The analysis of the experimental results reveals the following key findings. Shutters demonstrably mitigate building heat losses through facade window niches relative to unshuttered window configurations. The thermal resistance of shuttered systems exhibits a 1.5- to 2-fold increase, accompanied by a commensurate reduction in heat flux densities. Across all investigated configurations, the intrinsic thermal resistance of the 4M1-10-4M1-10-4M1 or 4M1i-10-4M1-10-4M1 double-glazed unit remained substantially constant. The implementation of an i-coating on the glazing substrate precipitates a further amplification of the thermal resistance in shuttered assemblies, culminating in a peak increment of 2 to 2.2 times. The integration of a single, stationary shutter panel, constructed from 2.0 mm thick flat polycarbonate, within the external compartment of the window reveal (environmental side) results in an average thermal resistance increment of 15.5 m^2^K/W.

The maximal increment in thermal resistance is attained when a shutter is deployed within the interior environment, co-planar with the internal wall surface, resulting in an augmentation approaching 50 m^2^K/W. The enhancement of thermal resistance in window assemblies equipped with shutters, coupled with the corresponding reduction in heat losses, is a direct consequence of the air interlayer formed between the shutters and the glazing. Consequently, the incorporation of shutters, regardless of their placement, markedly improves the energy performance of the window assembly and the overall building.

## 5. Conclusions

The development of accurate methods for calculating heat transfer through transparent structures is a critically important issue, since the specific heat loss through windows is many times higher than the heat loss through wall structures. The main function of windows is to transmit visible radiation into the building. Reducing heat loss (in winter) or excessive heat gain (in summer) is the second important goal of windows.

The underlying concept of this work involves the supplementary implementation of a vertical, thin, transparent barrier within the window reveal. Polycarbonate was chosen because it is actively used in the glass industry; in particular, it is used for conditionally explosion-proof laminated glass, which, under the influence of a shock wave, scatters into fewer fragments, and therefore injures people less. In Ukraine, this is a well-known experience from the realities of military life. A thickness of 2 mm represents the minimum available in serial production. Cellular polycarbonate with a thickness of 4 mm, also a commercially minimal dimension, was employed solely for experimental purposes. This material essentially comprises two thin vertical layers, each approximately 0.5 mm thick, separated by internal membranes. Its suitability is limited due to the non-planar, undulated surface of cellular polycarbonate sheets, which increases their area and results in the scattering of solar radiation, thereby reducing the amount of light transmitted into the building’s interior. This was shown by measurements with a luxmeter. A significant quantity of vertical polycarbonate chambers restricts the attainment of regulatory illumination within the premises, as windows primarily serve to admit visible radiation into the interior.

Initially, it is important to note that theoretical calculations conducted for external temperatures of 0 °C and −25 °C yielded a thermal resistance of the structure that remained essentially constant (within the margin of calculation error). That is, the results obtained in the article are suitable for both a moderate winter climate and significant frosts.

Regarding variations and the optimisation of the architectural design, we note that the studies were conducted for the opening in the facade wall of an experimental passive house, in which a window niche is arranged. It is for such architecture and composition of wall materials (Figure 2 and Figure 14) that experiments were conducted with a window block. For another basic window or for another facade wall design, similar studies should be conducted.

For practical application, a simple and economically affordable design can be recommended. For example, in a wooden U-shaped frame, several vertical (and horizontal for the lower lintel) parallel grooves with a depth of 2–3 cm are milled on its inner surface, into which thin sheets of transparent polycarbonate are tightly inserted from above. The optimal distance between the grooves is 4 cm. From above, such a design is covered with an upper board with corresponding grooves. The external dimensions of this design should correspond to the internal dimensions of the window niche. The structure is tightly mounted in the window niche and attached to the window in a way that is similar to the fastening of an anti-mosquito net. Leaks in the fit can be sealed with a foam cord or strip.

Compared to advanced multi-chamber double-glazed windows, our theoretical and experimental work suggests the following energy-efficient designs: (1) nanostructured transparent aerogel filling for inter-pane spaces, potentially increasing thermal resistance to 1.0–1.5 m^2^K/W, and (2) the sequential installation of two dual-chamber windows (separation > 10 cm), enhancing thermal resistance to 1.0 m^2^K/W. Vacuum glazing is another option. However, aerogel or double dual-chamber glazing are more costly than our proposed design, which achieves up to 1.8 m^2^K/W.

This study investigated the impact of external shutters on the thermal resistance of window assemblies under real meteorological conditions. Through a combination of numerical simulations and experimental measurements, the research demonstrated the significant potential of shutters to enhance the energy efficiency of buildings. The incorporation of external shutters substantially increased the thermal resistance of the window assemblies. Experimental data revealed a 1.5- to 2-fold increase in thermal resistance compared to unshuttered windows, accompanied by a corresponding reduction in heat flux densities. The number and placement of shutters significantly influenced overall thermal performance. In particular, the introduction of four shutters, strategically positioned within the window opening niche, nearly doubled the thermal resistance. Furthermore, the internal placement of the shutter resulted in the highest increase in thermal resistance. The utilisation of low-emissivity (i-coating) glass in conjunction with shutters further amplified the thermal resistance, achieving a maximum increase of 2 to 2.2 times. This highlights the effect of combining advanced glazing technologies with passive shading strategies. The improved thermal performance of shuttered window assemblies is attributed to the formation of an insulating air gap between the shutters and the glazing. This air gap effectively reduces the amount of conductive and convective heat transfer, thereby enhancing the overall thermal resistance. The findings of this study underscore the practical benefits of implementing external shutters as a cost-effective strategy to improve the energy efficiency of buildings. The straightforward installation and minimal financial investment associated with shutters make them a viable solution for both new and existing constructions.

## Figures and Tables

**Table 1:** Thermal resistance values for window assembly configurations.

Thermal Resistance, (m^2^K/W)	Var1	Var2	Var3
double-glazed unit (CFD Model)	0.33	0.34	0.32
double-glazed unit with shutters (CFD Model)	0.56	-	0.64
double-glazed unit with shutters and i-glass (CFD Model)	0.62	-	0.78
double-glazed unit with shutters and i-glass (Experiment (−2%))	0.61		
double-glazed unit with i-glass (CFD Model)	0.43	0.48	0.43
double-glazed unit with i-glass (Experiment (−2%))		0.49	

**Table 2:** Experimental thermal resistance parameters for the 4M1i-10-4M1-10-4M1 glazing unit with varied shutter arrangement.

Serial Number	Configuration Description	Thermal Resistance (m^2^K/W)	Percentage Increase in Thermal Resistance Relative to Standard Window	Increment in Thermal Resistance Relative to Preceding Configuration (m^2^K/W)
1	standard 4M1i-10-4M1-10-4M1 window (DSTU V B2.7-107:2008)	0.64	-	-
2	window only (experimental data: 8–10 November 2024)	0.63/0.64	-	-
3	window with one shutter (experimental data: 15–16 November 2024)	0.80	25	0.16
4	window with two shutters (experimental data: 20–22 November 2024)	0.99/0.97	53	0.18
5	window with three shutters (experimental data: 22–25 November 2024)	1.09/1.11/1.13	73	0.13
6	window with four shutters (experimental data: 25–27 November 2024)	1.27/1.25	97	0.15
7	shutter + window with four shutters (experimental data: 25 November–2 December 2024)	1.75/1.74/1.74/1.76	173	0.49

## Data Availability

The original contributions presented in this study are included in the article. Further inquiries can be directed to the corresponding author.
